# Supplementary material for: Pathology and genetics in a global cohort of Parkinsonian Disorders
Source: medRxiv. 2026 Mar 26:2026.03.23.26348322. Preprint. [Version 1] doi: 10.64898/2026.03.23.26348322 (PMC13042126; doi:10.64898/2026.03.23.26348322)
Supplement: Supplement 1 [file media-1.pdf]

Table of content

eMethods **2**

Study design 2

DNA extraction 3

Genotyping 3

Genome sequencing 3

Quantification of NeuroBooster array probe performance 4

eTables **5**

eTable 1. Number of clinically diagnosed movement disorder cases and control by brain bank 5

eTable 2. Correspondence between Lewy Body Pathology staging systems 6

eTable 3. List of genes assessed in short-read Genome Sequencing (n=38) 7

eTable 4. Variants identified from short-read Genome Sequencing (n=17) and additional variants of interest (n=21) analysed in genotyped samples 9

eTable 5. Frequency of primary pathological diagnosis by genetically determined ancestry 11

eTable 6. Distribution of incidental Lewy body cases by Lewy body disease stage 12

eTable 7. Distribution of secondary pathologies by primary pathology groups 12

eTable 8. Distribution of pathology stages by clinical diagnosis in Lewy body diseases 13

eTable 9. Frequency of GBA1 variants aggregated into Gaucher's Disease causing mutations group and Parkinson's Disease risk mutations group by pathological diagnosis 14

eTable 10. Cases with pathogenic LRRK2 mutations 14

eTable 11. Key Resource Table 15

eTable 12. Performance of NeuroBooster Array (NBA) probes compared with short-read Genome Sequencing (GS) for neurodegenerative movement disorder associated variants. 18

eTable 13. Variants with NeuroBooster Array (NBA) probes not detected in short-read Genome Sequencing (GS). 25

eFigure 1. Proportion of LBD and PSP diagnoses across ancestry groups. 26

References **27**

Pathology and genetics in a global cohort of Parkinsonian Disorders

eMethods

Study design

Understanding movement disorders through the integrated analysis of Genetics and neuroPathology (MD-GAP) includes 11 brain banks (eTable 1) and is funded by the Medical Research Council UK and Global Parkinson's Genetic Program (GP2, https://gp2.org/). We obtained ethical approval to coordinate the MD-GAP study from the UCL Queen Square Institute of Neurology research ethics committee (23473/001). Each brain bank has local ethics approval for the recruitment, storage and distribution of brain material from brain donors with appropriate consent. We set up data and material sharing agreements with each brain bank in the UK and Australia. An additional contract was signed with the Michael J. Fox Foundation (MJFF), allowing all genetic data generated through this project to be available on Verily Workbench (https://workbench.verily.com/), a cloud-native platform designed to host harmonised data for biomedical research.

We recruited all available subjects from collaborating brain banks with either DNA or frozen tissue for DNA extraction, with clinically or pathologically confirmed diagnoses of movement disorders. Clinical diagnoses include Parkinson's disease (PD), Parkinson's disease dementia (PDD), dementia with Lewy bodies (DLB), progressive supranuclear palsy (PSP), corticobasal syndrome (CBS), multiple system atrophy (MSA), and neurologically healthy controls. Pathological movement disorders diagnoses include Lewy body disorders (LBD), progressive supranuclear palsy, corticobasal degeneration (CBD), multiple system atrophy and Other (Alzheimer's disease, Argyrophilic grain disease, Cerebral amyloid angiopathy, Cerebral Vascular Disease, Chronic Traumatic Encephalopathy, Frontotemporal lobar degeneration, Primary Age Related Tauopathy, Pick's Disease, Tauopathy not otherwise specified, Tumour). Cases with a pathological diagnosis of motor neuron disease or prion disease were excluded from the study.

The summary demographic, clinical, and pathological data were obtained through three sources: direct sharing by the brain banks' manager, extraction from the UK Brain Bank Network (UKBBN) database, and clinical and pathology reports for cases from London Neurodegenerative Diseases Brain Bank (King's College London) and Multiple Sclerosis and Parkinson's Tissue Bank (Imperial College London). Clinical diagnoses were assigned according to these sources, and where available, clinical records were reviewed by MD-GAP's clinical team. Pathological diagnoses were recorded as reported in the pathology reports, preserving the original order of diagnoses. In cases where a formal diagnosis was not provided but semi-quantitative staging was available, a pathological diagnosis was inferred based on the staging data. Some cases had a clinical diagnosis but no corresponding pathological findings; for example, a clinical diagnosis of PD without LBs on Braak staging was recorded as a control. Cases were only recorded as lacking co-pathologies if this was explicitly stated in the reports. To improve transparency and facilitate assessment of inter-center variability, we compiled a table (eTable 12) summarising diagnostic criteria, staging system and antibodies used by the contributing brain banks, where such information was available.

We used REDCap, a secure web-based application designed for data collection and management (https://projectredcap.org/) to store clinical and pathological data. The database includes five instruments: 1) Base and demographics, 2) Family history, 3) Clinical information, 4) Neuropathology and 5) Genetics and transcriptomics. The data fields were harmonised with data fields from the GP2 data dictionary to facilitate data integration and large-scale meta-analyses. GP2 is an ASAP-funded project aimed at genotyping over 150,000 participants and genome sequence (GS) over 10,000 individuals across the world to further our understanding of the genetic architecture of PD. GP2 includes clinical and brain bank cohorts.

DNA extraction

DNA was extracted from either the cerebellum or frontal cortex. We conducted quality control by performing Qubit fluorometry or nanodrop (260/280 ratio of ~1.8) to determine DNA concentration and diluted accordingly in 96-well plates before genotyping and sequencing.

Genotyping

Available samples were genotyped using the Illumina Global Diversity Array chip with Neurobooster, which has a high-density SNV backbone (1.9 M SNVs) with an extra 95K custom content added, including known causal variants for various neurodegenerative diseases and imputation boosters for underrepresented populations (https://github.com/GP2code/Neuro_Booster_Array). Unlike GS, microarray genotyping focuses on specific regions. Genotyped data were called from IDAT files and processed using GenoTools (https://github.com/GP2code/GenoTools), a pipeline developed to automate the quality control (QC) process and estimation of ancestry. All data were imputed against the TOPMed r2 panel with Eagle v2.4 phasing on the TOPMed Imputation Server using Minimac4 (https://imputation.biodatacatalyst.nhlbi.nih.gov/33).

Genotype data were filtered using PLINK v2 (https://www.cog-genomics.org/plink/2.0/). Individuals and SNPs with >5% missing data were excluded using the –mind 0.05 and –geno 0.05 filters, respectively, to ensure high-quality data.

Variant selection was based on GS findings and additional important movement disorder-associated variants, shown in eTable 4. GS identified variants from this cohort (n=17) classified as pathogenic/ likely pathogenic based on Clinvar (https://www.ncbi.nlm.nih.gov/clinvar/ downloaded on 2024/09/17) were assessed in all cases that underwent NeuroBooster array (NBA). Of the 17 variants detected by GS, 13 had valid probes in the Illumina NBA chip. Additional rare variants (n = 21) of interest in *GBA1*, *LRRK2*, *PINK1*, *PRKN*, *SNCA* and *VPS35* with probes present in the NBA chip were selected. This includes all *LRRK2* and *SNCA* variants defined as pathogenic in Genereviews (https://www.ncbi.nlm.nih.gov/books/NBK1116/) and ClinVar (https://www.ncbi.nlm.nih.gov/clinvar/), *GBA1* variants (PD risk alleles and GD causing variants) that occurred in >0.1% of European PD as per Malek et al. 2018 [1], *PRKN* variants with >1% frequency in autosomal recessive PD patients as per Menon et al. 2024 [2], and the *PINK1* L347P variant described by Morales-Briceno et al. [3]. Therefore, a total of 34 variants were assessed in cases that underwent NBA.

Genome sequencing

Selected samples (n = 2954) underwent Illumina short-read Genome sequencing (GS) to examine both coding and non-coding regions, enabling detection of rare and common variants, structural variations, and mutations linked to phenotypes. Reads were aligned to the GRCh38DH reference genome, and small variants were called.

Joint genotyping was performed using the Broad Institute's pipeline for samples passing AMP-PD quality control (https://amp-pd.org). gVCFs generated with DeepVariant were merged using GLnexus (v1.6.1) with settings requiring minimum allele quality scores (min_AQ1, min_AQ2 ≥10), allowing monoallelic sites, and supporting up to 32 alternate alleles. The pipeline preserved partial data, genotype likelihoods (PL), and revised genotypes. Sample-level FORMAT fields—DP, AD, GQ, and PL—were conservatively merged (e.g., minimum DP and GQ values).

High-quality variants were retained if they passed variant quality score recalibration, had a call rate >0.95, genotype quality ≥20 and read depth ≥10. No allele balance (AB) filtering was applied at the sample level. Samples were excluded for excessive heterozygosity (F-statistic > ±0.15) or sex mismatches between clinical and genetic sex (based on the X chromosome). Relatedness checks were used to identify duplicates and contamination.

A total of 415 genes were selected based on the Genomics England PanelApp Adult Onset Neurodegenerative Disorders panel, release R.58 (v7.15, 30 October 2024) https://panelapp.genomicsengland.co.uk/panels/474/. Gene-level variant extraction was performed using bcftools (bcftools(1)) and variant-level annotation was performed with ANNOVAR (December 2023, ANNOVAR Documentation). Only genes associated with a movement disorder phenotype and a "green" (GEL status 3) classification, indicating a high level of evidence supporting the gene's role in disease, were included in the final analysis (n=36). Exceptions were made for genes of particular interest

regardless of GEL status, including RAB39B and VPS13C, therefore, 38 genes were examined as described in eTable 3.

Variants in dominant and recessive genes were retained if coding or splicing, and classified as pathogenic/ likely pathogenic according to Clinvar (https://www.ncbi.nlm.nih.gov/clinvar/). *GBA1* variants were retained irrespective of Clinvar classification and allele frequency. Single heterozygous carriers in recessive genes were excluded from analysis, except for *GBA1*. Synonymous variants were excluded.

17 variants of interest were identified in 2954 cases, shown in eTable 4.

Quantification of NeuroBooster array probe performance

In a subset of cases (n=2214) across varied cohorts both NBA and GS data were available. We calculated the proportion of false positive and false negative genotype calls and calculated the sensitivity and specificity of each probe for variants detected by NBA, considering GS as the reference (eTable 13). When more than one probe was available for the same variant, the best probe was selected, prioritising specificity over sensitivity to limit the rate of false positives. Where probes performed equally, a single probe per variant was selected. Of the variants searched for in NBA, 21 probes were validated against GS, each demonstrating a specificity greater than 99.9%.

eTable 14 lists NBA probe names for variants not detected in GS. As these variants were not observed in GS, their corresponding probes were not validated and were not reported further.

Combining GS and validated findings genotyping, 20 unique mutations were identified in our cohort. Following collaboration with brain banks and review of relevant literature, variants in *PSAP* and *C19orf12* were excluded from the main results shown in Table 3. In contrast to the classification in PanelApp, we found no confirmatory segregation studies for *PSAP*, and reported cases have largely involved sporadic rather than familial disease [4]. Furthermore, neither of the two cases with *C19orf12* variants demonstrated brain iron accumulation on post-mortem examination, questioning its pathogenicity. Accordingly, only 18 pathogenic or likely pathogenic variants are reported in the main text.

eTables

eTable 1. Number of clinically diagnosed movement disorder cases and control by brain bank

Brain Bank	Contacts	N
Queen Square Brain Bank	**Zane Jaunmuktane**<br>**Tammaryn Lashley**<br>**Tom Warner**	1335
Multiple Sclerosis and Parkinson's Tissue Bank	Djordje Gveric<br>**Steve Gentleman**	637
Banner Sun Health Research Institute [5]	**Thomas Beach**<br>Geidy Serrano	551
Edinburgh Brain and Tissue Bank	**Colin Smith**<br>Chris-Anne McKenzi	138
Newcastle Brain Tissue Resource	Debbie Lett<br>**Chris Morris**	113
Oxford Brain Bank	Carolyn Sloan<br>**Laura Parkkinen**	116
Sydney Brain Bank	**Glenda Halliday**<br>**Claire Shepherd**	110
Manchester Brain Bank	**Andrew Robinson**<br>**Federico Roncaroli**	110
London Neurodegenerative Diseases Brain Bank	Claire Troakes<br>**Andrew King**	101
South West Dementia Brain Bank	Candida Tasman<br>Richard Cain<br>**Seth Love**	78
Victoria Brain Bank	**Catriona McLean**	64
**Total**	-	3353

11 brain banks have been included in the present study. UK and Australian brain banks are part of the MD-GAP study. Banner Sun Health Research Institute is part of the GP2 study only.
In the Contacts column, group Principal Investigators are in bold

eTable 2. Correspondence between Lewy Body Pathology staging systems

Unified staging system for LBD	McKeith stage	Lewy Body Braak stage
Neocortical	Neocortical	Stages 5 - 6
Limbic	Limbic	Stage 4
Brainstem	Brainstem	Stages 1 - 3
Amygdala	Amygdala	..

A table showing equivalence between different staging systems. Based on BrainNet Europe Protocol [6].

eTable 3. List of genes assessed in short-read Genome Sequencing (n=38)

Gene	Mode of Inheritance	Phenotypes
ATP1A3	Monoallelic	Alternating hemiplegia of childhood; CAPOS syndrome; Rapid-Onset Dystonia-Parkinsonism
AUH	Biallelic	Dystonia
C19orf12	Both Monoallelic and Biallelic	Spastic paraplegia; Neurodegeneration with brain iron accumulation
CHCHD2	Monoallelic	PD
CHMP2B	Monoallelic	FTD and/or amyotrophic lateral sclerosis; Dystonia
COASY	Biallelic	COASY protein-associated neurodegeneration; Neurodegeneration with brain iron accumulation
DNAJC6	Biallelic	EOPD
FBXO7	Biallelic	Dystonia; PD
FTL	Monoallelic	Neurodegeneration with brain iron accumulation
GBA	Biallelic	Late-onset PD
GCH1	Both Monoallelic and Biallelic	Dystonia; DOPA-responsive; with or without hyperphenylalaninemia
KIAA1161	Biallelic	Basal ganglia calcification
LRRK2	Monoallelic	PD
LYST	Biallelic	Chediak-Higashi syndrome; peripheral neuropathy; Parkinsonism; spastic paraplegia
MAPT	Monoallelic	FTD with or without parkinsonism
NAA60	Biallelic	Basal ganglia calcification
NPC1	Biallelic	Niemann-Pick disease
NPC2	Biallelic	Dystonia; Niemann-Pick disease
PANK2	Biallelic	Dystonia; Neurodegeneration with brain iron accumulation

PARK7	Biallelic	EOPD
PDGFB	Monoallelic	Basal ganglia calcification
PDGFRB	Monoallelic	Dystonia; Basal ganglia calcification
PINK1	Biallelic	EOPD; Dystonia
**Gene**	**Mode of Inheritance**	**Phenotypes**
PLA2G6	Biallelic	PD; Neurodegeneration with brain iron accumulation
PRKN	Biallelic	PD
PSAP	Monoallelic	PD
RAB32	Monoallelic	PD
RAB39B	X-Linked: hemizygous mutation in males, monoallelic mutations in females may cause disease (may be less severe, later onset than males)	Early onset parkinsonism and intellectual disability; Waisman syndrome
SLC20A2	Monoallelic	Dystonia; Basal ganglia calcification
SNCA	Monoallelic	PD; Dementia
SPG11	Biallelic	Early onset parkinsonism; Spastic paraplegia
SYNJ1	Biallelic	PD
TBK1	Monoallelic	FTD and/or ALS; PSP-like and Cerebellar phenotypes
TREM2	Biallelic	Polycystic lipomembranous osteodysplasia with sclerosing leukoencephalopathy; Dystonia
VPS13C	Biallelic	EOPD
VPS35	Monoallelic	PD
WDR45	X-Linked: hemizygous mutation in males	Dystonia;Neurodegeneration with brain iron accumulation
XPR1	Monoallelic	Basal ganglia calcification

Abbreviations: PD: Parkinson disease; EOPD: Early onset Parkinson disease; LOPD: Late onset Parkinson disease; FTD: Frontotemporal dementia; ALS: Amyotrophic lateral sclerosis; PSP: Progressive Supranuclear Palsy

eTable 4. Variants identified from short-read Genome Sequencing (n=17) and additional variants of interest (n=21) analysed in genotyped samples

Gene	Hg38 Chr:pos:ref:alt	HGVS	NBA Illumina probe present (Y/N)	Variant selection
C19orf12	19:29702957:29702967:ACAGCCCCCC G:-	p.Gly58ArgfsTer10	N	Short-read GS
GBA1	1:155235843:T:C	p.Asn409Ser	Y	Short-read GS
GBA1	1:155236246:G:A	p.Thr408Met	Y	Short-read GS
GBA1	1:155236376:C:T	p.Glu365Lys	Y	Short-read GS
GBA1	1:155238192:A:G	p.Ser235Pro	Y	Short-read GS
GBA1	1:155237444:A:G	p.Ile299Thr	Y	Short-read GS
GBA1	1:155235252:A:G	p.Leu483Pro	Y	Short-read GS
GBA1	1:155237426:G:-	p.Pro305LeufsTer 31	N	Short-read GS
GBA1	1:155238630:G:A	p.Arg159Trp	N	Short-read GS
GBA1	1:155235727:C:G	p.Asp448His	Y	Short-read GS
GBA1	1:155240629:C:T	..	Y	Short-read GS
GBA1	1:155236384:G:A	p.Thr362Ile	Y	Short-read GS
LRRK2	12:40340400:G:A	p.Gly2019Ser	Y	Short-read GS
LRRK2	12:40321114:A:G	p.Tyr1699Cys	Y	Short-read GS
PRKN	6:161785820:G:A	p.Arg275Trp	Y	Short-read GS
PSAP	10:71851221:T:A	p.Met1Leu	Y	Short-read GS
TBK1	12:64498008:G:T	p.Glu703Ter	N	Short-read GS
GBA1	1:155235196:G:A	p.Arg502Cys	Y	Additional variant of interest

GBA1	1:155238597:G:A	p.Arg170Cys	Y	Additional variant of interest
PINK1	1:20645640:T:C	p.Leu347Pro	Y	Additional variant of interest
**Gene**	**Hg38 Chr:pos:ref:alt**	**HGVS**	**NBA Illumina probe present (Y/N)**	**Variant selection**
PRKN	1:34784887:G:A	p.Arg42Pro	Y	Additional variant of interest
LRRK2	12:40299125:A:G	p.Ile1122Val	Y	Additional variant of interest
LRRK2	12:40309225:A:G	p.Asn1437Asp	Y	Additional variant of interest
LRRK2	12:40310434:C:T	p.Arg1441Cys	Y	Additional variant of interest
LRRK2	12:40310435:G:A	p.Arg1441His	Y	Additional variant of interest
LRRK2	12:40340404:T:C	p.Ile2020Thr	Y	Additional variant of interest
LRRK2	12:40363526:G:A	p.Gly2385Asp	Y	Additional variant of interest
VPS35	16:46662452:C:T	p.Asp620Asn	Y	Additional variant of interest
SNCA	4:89828149:C:T	p.Ala53Thr	Y	Additional variant of interest
SNCA	4:89828154:C:T	p.Gly51Asp	Y	Additional variant of interest
SNCA	4:89828170:C:T	p.Glu46Lys	Y	Additional variant of interest
SNCA	4:89835580:C:G	p.Ala30Pro	Y	Additional variant of interest
PRKN	6:161350205:C:T	p.Cys431Phe	Y	Additional variant of interest
PRKN	6:161350208:C:T	p.Gly430Asp	Y	Additional variant of

Gene	Hg38 Chr:pos:ref:alt	HGVS	NBA Illumina probe present (Y/N)	Variant selection
				interest
PRKN	6:161360190:G:T	p.Glu395Ter	Y	Additional variant of interest
PRKN	6:161785885:C:T	p.Cys253Tyr	Y	Additional variant of interest
PRKN	6:162262715:C:CCA	p.Trp74CysfsTer8	Y	Additional variant of interest
PRKN	6:162443378:CCT:C	p.Gln34ArgfsTer5	Y	Additional variant of interest

eTable 4 contains all neurodegenerative movement disorder associated variants analysed in genotyped samples. Variants were selected from two sources: (1) ClinVar pathogenic/likely pathogenic variants identified from short-read genome sequencing (GS) (n=17; 13 with valid Illumina NBA chip probes), and (2) additional rare variants of interest (n=21) in *GBA1, LRRK2, PINK1, PRKN, SNCA,* and *VPS35.* A total of 34 variants were evaluated in NBA-genotyped cases.

eTable 5. Frequency of primary pathological diagnosis by genetically determined ancestry

	AAC/AFR	AJ	AMR	CAH	CAS	EAS	EUR	MDE	SAS
**N***	5	63	8	9	1	3	2758	2	18
**Sex, F (%)**	1 (20.0%)	25 (39.7%)	3 (37.5%)	3 (33.3%)	0 (0.0%)	2 (66.7%)	1051 (38.1%)	1 (50.0%)	8 (44.4%)
**Age at death, years(sd)**	71.2 (3.7)	79.9 (9.1)	73.8 (12.7)	73.2 (9.0)	53	72.7 (1.5)	77.2 (10.2)	81.5 (7.8)	71.1 (4.9)
**Pathology**									
LBD	3 (60.0%)	40 (63.5%)	1 (12.5%)	1 (11.1%)	0 (0.0%)	1 (33.3%)	1510 (54.7%)	1 (50.0%)	2 (11.1%)
PSP	2 (40.0%)	5 (7.9%)	1 (12.5%)	5 (55.6%)	0 (0.0%)	1 (33.3%)	433 (15.7%)	1 (50.0%)	12 (66.7%)
CBD	0 (0.0%)	0 (0.0%)	1 (12.5%)	0 (0.0%)	0 (0.0%)	1 (33.3%)	14 (0.5%)	0 (0.0%)	0 (0.0%)
MSA	0 (0.0%)	3 (4.8%)	0 (0.0%)	0 (0.0%)	1 (100.0%)	0 (0.0%)	187 (6.8%)	0 (0.0%)	2 (11.1%)

Control	0 (0.0%)	13 (20.6%)	4 (50.0%)	2 (22.2%)	0 (0.0%)	0 (0.0%)	518 (18.8%)	0 (0.0%)	1 (5.5%)
Other	0 (0.0%)	2 (3.2%)	1 (12.5%)	1 (11.1%)	0 (0.0%)	0 (0.0%)	96 (3.5%)	0 (0.0%)	1 (5.5%)

AAC:African American, AFR:African, AJ:Ashkenazi Jews, AMR:Admixed American/Latin American, CAH:Complex Admixture History, CAS:Central Asian, EAS:East Asian, EUR: European, MDE: Middle Eastern, SAS: South Asian
LBD: Lewy Body Diseases,PSP: Progressive Supranuclear Palsy, MSA: Multiple System Atrophy, CBD: Corticobasal Degeneration

eTable 6. Distribution of incidental Lewy body cases by Lewy body disease stage

LBD Subtype	N	N with *APOE* e4
Amygdala	1 (3.0%)	0 (0.0%)
Brainstem	7 (21.2%)	0 (0.0%)
Limbic	7 (21.2%)	0 (0.0%)
Neocortical	10 (30.3%)	2 (40.0%)
No information available	5 (15.2%)	1 (20.0%)
*Unclassifiable	3 (9.1%)	2 (40.0%)
Total	33	5 (15.2%)

*Cases listed as “Unclassifiable” were designated as such according to the Braak LB system

eTable 7. Distribution of secondary pathologies by primary pathology groups

		Primary Pathology			
		LBD	PSP	MSA	CBD
	n*	1064	216	20	12
Secondary Pathology	None	209 (19.6%)	1 (0.5%)	0 (0.0%)	0 (0.0%)

	**LBD**	..	40 (18.5%)	4 (20.0%)	3 (25.0%)
	**AD**	426 (40.0%)	35 (16.2%)	4 (20.0%)	2 (16.7%)
	**PSP**	13 (1.2%)	..	0 (0.0%)	0 (0.0%)
	**MSA**	5 (0.5%)	0 (0.0%)	..	0 (0.0%)
	**CBD**	3 (0.3%)	4 (1.9%)	0 (0.0%)	..
	**Other**	408 (38.3%)	136 (63.0%)	12 (60.0%)	7 (58.3%)

* This table presents only cases for which data on secondary pathology were available.
Other tauopathies: Argyrophilic grain disease, Chronic Traumatic Encephalopathy, Primary Age Related Tauopathy, Picks, Tauopathy, Aging-related tau astrogliopathy
Other: Cerebral amyloid angiopathy, Other, Limbic-predominant age-related TDP-43 encephalopathy, Frontotemporal Lobar Degeneration, tumour

eTable 8. Distribution of pathology stages by clinical diagnosis in Lewy body diseases

		PD	PDD	DLB
McKeith Stages	**Total**	744	288	140
	**None**	14 (1.9%)	2 (0.7%)	3 (2.1%)
	**Amygdala**	6 (0.8%)	3 (1.0%)	0 (0.0%)
	**Brainstem**	128 (17.2%)	48 (16.7%)	14 (10.0%)
	**Limbic**	200 (26.9%)	41 (14.2%)	10 (7.1%)
	**Neocortical**	396 (53.2%)	194 (67.4%)	113 (80.7%)
Braak LB	**Total**	582	237	80

	**0**	32 (5.4%)	7 (3.0%)	2 (2.5%)
	**1-2**	14 (2.5%)	3 (1.2%)	0 (0.0%)
	**3-4**	149 (25.6%)	27 (11.4%)	2 (2.5%)
	**5-6**	387 (66.5%)	200 (84.4%)	76 (95.0%)
**Braak NFT**	**Total**	778	351	178
	**0**	55 (7.1%)	18 (5.1%)	5 (2.8%)
	**I-II**	404 (51.9%)	187 (53.3%)	63 (35.4%)
	**III-IV**	258 (33.2%)	118 (33.6%)	82 (46.1%)
	**V-VI**	61 (7.8%)	28 (8.0%)	28 (15.7%)
**CERAD**	**Total**	703	258	123
	**No neuritic plaques**	388 (55.2%)	133 (51.6%)	25 (20.3%)
	**Sparse neuritic plaques**	119 (16.9%)	64 (24.8%)	16 (13.0%)
	**Moderate neuritic plaques**	113 (16.1%)	46 (17.8%)	28 (22.8%)
	**Frequent neuritic plaques**	83 (11.8%)	15 (5.8%)	54 (43.9%)
**Thal**	**Total**	615	248	121
	**0**	159 (26.0%)	77 (31.1%)	17 (14.0%)
	**1-2**	197 (32.0%)	69 (27.8%)	21 (17.4%)
	**3**	151 (24.5%)	55 (22.2%)	50 (41.3%)

	4-5	108 (17.5%)	47 (18.9%)	33 (27.3%)

Percentages are calculated using cases with available data for each staging system.
PD: Parkinson's Disease, PDD: Parkinson's Disease Dementia, DLB: Dementia with Lewy Bodies

eTable 9. Frequency of *GBA1* variants aggregated into Gaucher's Disease causing mutations group and Parkinson's Disease risk mutations group by pathological diagnosis

	LBD	PSP	CBD	MSA	Other	All Cases	Control	TOTAL
WGS N	1157	473	20	183	76	1909	387	2296
GBA1 GD variants	33 (2.9%)	1 (0.2%)	0	1 (0.5%)	0	35 (1.8%)	2 (0.5%)	37 (1.6%)
GBA1 Risk variants	131 (11.3%)	27 (5.7%)	1 (5.0%)	9 (4.9%)	5 (6.6%)	173 (9.1%)	33 (8.5%)	206 (9.0%)

LBD: Lewy Body Diseases,PSP: Progressive Supranuclear Palsy, MSA: Multiple System Atrophy, CBD: Corticobasal Degeneration
Other: Alzheimer's disease, Argyrophilic grain disease, Cerebral amyloid angiopathy, Cerebral Vascular Disease, Chronic Traumatic Encephalopathy, Frontaltemporal lobar degeneration, Primary Age Related Tauopathy, Pick's Disease, Small Vessels Disease, Tauopathy, Tumour
GBA1 GD causing mutations include: D448H, I299T, L483P, N409S, P305Lfs*31, R159W, R170C, R502C, S235P, T362I. GBA1 PD risk mutations include: E365K and T408M.

eTable 10. Cases with pathogenic *LRRK2* mutations

Case	Sex	Genetic ancestry	Clinical Diagnosis	Pathological Diagnosis	Age at death	Mutation
1	M	EUR	PD	LBD	81 - 85	G2019S
2	F	AJ	PD	LBD	81 - 85	G2019S
3	F	EUR	PD	PSP	91 - 95	G2019S
4	F	EUR	PD	Control	76 - 80	G2019S
5	F	EUR	PD	LBD	71 - 75	G2019S
6	M	EUR	PD	LBD	76 - 80	G2019S

7	F	AJ	PD	LBD	81 - 85	G2019S
8	M	EUR	PSP	FTLD	71 - 75	G2019S
9	M	CAH	PD	LBD	55 – 60	G2019S
10	F	EUR	PD	PSP	81 - 85	G2019S
11	F	EUR	PD	LBD	81 - 85	G2019S
12	F	EUR	PDD	TDP-43	86 - 90	G2019S
13	F	AJ	PD	LBD	81 - 85	G2019S
14	F	EUR	PD	Other	71 - 75	Y1699C
15	M	AJ	PD	LBD	61 - 65	G2019S
16	F	EUR	PD	LBD	81 - 85	G2019S
17	F	EUR	PD	LBD	71 - 75	G2019S
18	M	EUR	PD	LBD	71 - 75	G2019S
19	F	EUR	PD	LBD	86 - 90	G2019S

eTable 11. Key Resource Table

RESOURCE TYPE	RESOURCE NAME	SOURCE	IDENTIFIER	NEW/REUSE	ADDITIONAL INFORMATION
Dataset	Defining and Diagnosing neurodegenerative Movement Disorders through integrated analysis of Genetics and neuroPathology (MD-GAP)/Global Parkinson's	AMP-PD	DOI: 10.1002/mds.28494	NEW	All data generated in MD-GAP are now part of GP2. Full data can be accessed here: https://amp-pd.org/register-for-amp-pd

	Genetics Program (GP2)				
Software/code	R code: Analysis Script	Github	https://github.com/huw-morris-lab/MD-GAP-GP2-CPC.git	NEW	
Software/code	GenoTools	https://github.com/dvitale199/GenoTools	https://doi.org/10.1093/g3journal/jkae268	REUSE	
Software/code	R Project for Statistical Computing, v4.3.1	https://www.r-project.org/	RRID:SCR_001905	REUSE	
Software/code	R package: dplyr	https://cran.r-project.org/web/packages/dplyr/index.html	RRID:SCR_016708	REUSE	
Software/code	R package: data.table	https://github.com/Rdatatable/data.table	RRID:SCR_026117	REUSE	
Software/code	R package: readxl	https://cran.r-project.org/web/packages/readxl/index.html	RRID:SCR_018083	REUSE	
Software/code	R package: stringr	https://stringr.tidyverse.org/	RRID:SCR_022813	REUSE	
Software/code	R package: ggplot2	https://cran.r-project.org/web/packages/ggplot2/index.html	RRID:SCR_014601	REUSE	
Software/code	R package: scales	https://CRAN.R-project.org/package=scales	RRID:SCR_019295	REUSE	
Software/code	R package: RColorBrewer	https://cran.r-project.org/web/packages/RColorBrewer/index.html	RRID:SCR_016697	REUSE	
Software/code	R package: tidyverse	https://CRAN.R-	RRID:SCR_019186	REUSE	

		project.org/package=tidyverse			
Software/code	R package: pheatmap	https://www.rdocumentation.org/packages/pheatmap/versions/0.2/topics/pheatmap	RRID:SCR_016418	REUSE	
Software/code	R package: gridExtra	https://CRAN.R-project.org/package=gridExtra	RRID:SCR_025249	REUSE	
Software/code	R package: MASS	https://CRAN.R-project.org/package=MASS	RRID:SCR_019125	REUSE	
Software/code	R package: survival	https://CRAN.R-project.org/view=Survival	RRID:SCR_026244	REUSE	
Software/code	R package: survminer	https://rdocumentation.org/packages/survminer/versions/0.4.9	RRID:SCR_021094	REUSE	
Software/code	R package: broom	https://cran.r-project.org/web/packages/broom/index.html	RRID:SCR_026874	REUSE	
Software/code	Plink2	https://www.cog-genomics.org/plink/2.0/	RRID:SCR_001757	REUSE	

List of resources generated and used for this project.

eTable 12. Performance of NeuroBooster Array (NBA) probes compared with short-read Genome Sequencing (GS) for neurodegenerative movement disorder associated variants.

Gene	HGVS	Illumina probe name	TP	TN	FP	FN	Sensitivity	Specificity
GBA1	p.Asp448His	**1:155205518-G-C**	0	2211	0	3	0	1
GBA1	p.Asp448His	ilmnseq_rs1064651	0	2211	0	3	0	1
GBA1	p.Asp448His	ilmnseq_rs1064651_ilmndup2_ilmnbot	0	2211	0	3	0	1
GBA1	p.Asp448His	rs1064651	0	2211	0	3	0	1
GBA1	p.Glu365Lys	**chr1:155236376:C:T**	93	2119	1	1	0.989361702	0.999528302
GBA1	p.Glu365Lys	kgp308887	93	2119	1	1	0.989361702	0.999528302
GBA1	p.Ile299Thr	seq-rs794727908	0	2213	1	0	-	0.999548329
GBA1	p.Ile299Thr	**Seq_rs794727908**	0	2214	0	0	-	1

GBA1	p.Asn409Ser	exm106217	12	2199	1	2	0.8571 42857	0.9995 45455
GBA1	p.Asn409Ser	ilmnseq_rs76763715.1_F2BT	9	2199	1	5	0.6428 57143	0.9995 45455
GBA1	p.Asn409Ser	ilmnseq_rs76763715.2_F2BT	0	2200	0	14	0	1
GBA1	p.Asn409Ser	Seq_rs76763715.2_ilmnfwd_il mnF2BT	0	2200	0	14	0	1
GBA1	p.Asn409Ser	**chr1:155235843:T:C**	14	2198	2	0	1	0.9990 90909
GBA1	p.Arg170Cys	**chr1:155208388-155208388_G_T**	0	2212	0	2	0	1
GBA1	p.Arg170Cys	rs398123530	0	2212	0	2	0	1
GBA1	p.Arg502Cys	**1:155204987-C-T**	4	2210	0	0	1	1
GBA1	p.Arg502Cys	Seq_rs80356771.1_ilmnrev_il mnF2BT	4	2210	0	0	1	1

GBA1	p.Arg502Cys	Seq_rs80356771.2_ilmnrev_ilmnF2BT	0	2210	0	4	0	1
GBA1	p.Arg502Cys	rs80356771	4	2210	0	0	1	1
GBA1	c.115+1G>A	**rs104886460**	0	2213	0	1	0	1
GBA1	p.Thr362Ile	**1:155206175-C-T**	1	2213	0	0	1	1
GBA1	p.Thr362Ile	rs76539814	0	2213	0	1	0	1
GBA1	p.Thr362Ile	Seq_rs76539814	1	2213	0	0	1	1
GBA1	p.Thr408Met	exm106220	42	2167	2	3	0.933333333	0.999077916
GBA1	p.Thr408Met	rs75548401	39	2167	2	6	0.866666667	0.999077916
GBA1	p.Thr408Met	**seq_rs75548401**	43	2167	2	2	0.955555556	0.999077916
LRRK2	p.Gly2019Ser	**exm994671**	12	2202	0	0	1	1

LRRK2	p.Gly2019Ser	rs34637584	12	2202	0	0	1	1
LRRK2	p.Gly2019Ser	seq_rs34637584	12	2202	0	0	1	1
LRRK2	p.Gly2385Asp	**exm994721**	0	2214	0	0	-	1
LRRK2	p.Gly2385Asp	rs34778348	0	2214	0	0	-	1
LRRK2	p.Gly2385Asp	seq_rs34778348	0	2214	0	0	-	1
LRRK2	p.Arg1441His	**12:40704237-G-A**	0	2214	0	0	-	1
LRRK2	p.Arg1441His	Seq_rs34995376	0	2214	0	0	-	1
LRRK2	p.Tyr1699Cys	**12:40714916-A-G**	1	2213	0	0	1	1
LRRK2	p.Tyr1699Cys	Seq_rs35801418	1	2213	0	0	1	1

PRKN	p.Cys253Tyr	**PARK2:NM_004562.2:c.758 G>T:p.(Cys253Phe)**	0	2214	0	0	-	1
PRKN	p.Gly430Asp	**Seq_rs191486604**	2	2212	0	0	1	1
PRKN	p.Gly430Asp	exm593474	2	2212	0	0	1	1
PRKN	p.Asn52Metf sTer24	**Variant13588**	1	2213	0	0	1	1
PRKN	p.Asn52Metf sTer24	Seq_rs754809877	1	2213	0	0	1	1
PRKN	p.Arg275Trp	exm593505	19	2194	0	1	0.95	1
PRKN	p.Arg275Trp	**rs34424986**	20	2194	0	0	1	1
PRKN	p.Arg275Trp	Seq_rs34424986.1_ilmnrev_il mnF2BT	6	2194	0	14	0.3	1
PRKN	p.Arg275Trp	Seq_rs34424986.2_ilmnrev_il mnF2BT	0	2194	0	20	0	1
PRKN	p.Trp74Cysfs	**indel.94051**	1	2213	0	0	1	1

	Ter8							
PSAP	p.Met1Leu	**10:73610978-A-T**	1	2213	0	0	1	1
PSAP	p.Met1Leu	Seq_rs121918106.1_ilmnrev_ilmnF2BT	1	2213	0	0	1	1
PSAP	p.Met1Leu	Seq_rs121918106.2_ilmnrev_ilmnF2BT	0	2212	1	1	0	0.999548125
SNCA	p.Gly51Asp	**seq-rs431905511-B1**	1	2213	0	0	1	1
VPS35	p.Asp620Asn	**16:46696364-G-A**	0	2214	0	0	-	1

The proportion of true positive (TP), true negative (TN), false positive (FP) and false negative (FN) genotype calls, and probe sensitivity and specificity, were calculated for the 51 probes targeting mutations detected in the 2214 individuals who underwent both NeuroBooser Array (NBA) genotyping and short-read Genome Sequencing. The most accurate probes were selected for analysis, shown in bold.

eTable 13. Variants with NeuroBooster Array (NBA) probes not detected in short-read Genome Sequencing (GS).

Gene	HGVS	Illumina probe name
GBA1	p.Leu483Pro	1:155205043-T-C
GBA1	p.Ser235Pro	Variant49129
LRRK2	p.Ile1122Val	12:40692927-A-G
LRRK2	p.Ile1122Val	Seq_rs34805604
LRRK2	p.Ile2020Thr	12:40734206-T-C
LRRK2	p.Ile2020Thr	Seq_rs35870237
LRRK2	p.Asn1437Asp	12:40703027-A-C
LRRK2	p.Asn1437Asp	Seq_rs74163686
LRRK2	p.Arg1441Cys	Seq_rs33939927.1_ilmnfwd_ilmnF2BT
LRRK2	p.Arg1441Cys	Seq_rs33939927.2_ilmnfwd_ilmnF2BT
LRRK2	p.Arg1441Cys	Seq_rs33939927.3_ilmnfwd_ilmnF2BT
LRRK2	p.Arg1441Cys	var_12_40704236
PINK1	p.Leu347Pro	1:20972133-T-C
PINK1	p.Leu347Pro	Seq_rs28940285
PINK1	p.Leu347Pro	rs28940285
PRKN	p.Gln34ArgfsTer5	Variant17166
PRKN	p.Cys431Phe	6:161771237-G-T
PRKN	p.Glu395Ter	PARK2:NM_004562.2:c.1183G>T:p.(Glu395*)
PRKN	p.Arg42Pro	1:35250488-G-C

SNCA	p.Ala30Pro	rs104893878
SNCA	p.Ala53Thr	seq-rs104893877-B1
SNCA	p.Glu46Lys	4:90749321-G-A

A table listing additional variants of interest and corresponding Illumina NeuroBooster Array (NBA) probe names. These variants were not identified in short-read Genome Sequencing, hence probe performance could not be assessed.

eFigures

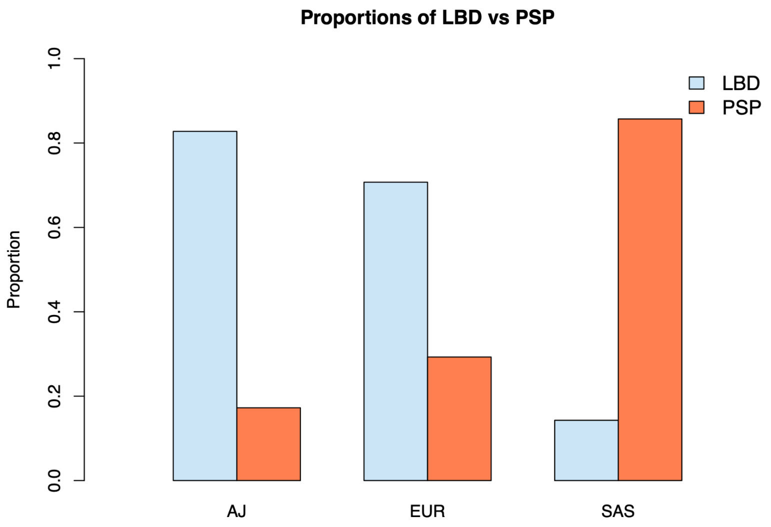

eFigure 1. Proportion of LBD and PSP diagnoses across ancestry groups.

Bar plot showing the relative proportion of Lewy body disease (LBD) and progressive supranuclear palsy (PSP) in individuals of Ashkenazi Jewish (AJ), European (EUR) and South Asian (SAS) ancestry. Mutation carriers were excluded from this analysis to avoid confounding effects from *GBA1* and *LRRK2*, which are known to be enriched in AJ. PSP was more frequent in SAS compared with EUR and AJ, while LBD was more frequent in AJ ($p < 0.0001$).

References

1. Malek N, Weil RS, Bresner C, et al. Features of GBA-associated Parkinson’s disease at presentation in the UK Tracking Parkinson's study. *J Neurol Neurosurg Psychiatry*. 2018;89(7):702-709.

2. Menon PJ, Sambin S, Criniere-Boizet B, et al. Genotype-phenotype correlation in PRKN-associated Parkinson’s disease. *NPJ Parkinsons Dis*. 2024;10(1):72.

3. Morales-Briceno H, Ong TL, Duma SR, et al. Recurrent biallelic p.L347P PINK1 variant in Polynesians with parkinsonism and isolated dopa-responsive dystonia. *Mov Disord Clin Pract*. 2022;9(5):696-697.

4. Chen YP, Gu XJ, Ou RW, et al. Genetic analysis of prosaposin, the lysosomal storage disorder gene in Parkinson’s disease. *Mol Neurobiol*. 2021;58(4):1583-1592.

5. Beach TG, Adler CH, Sue LI, et al. Arizona Study of Aging and Neurodegenerative Disorders and brain and Body Donation Program. *Neuropathology*. 2015;35(4):354-389.

6. Alafuzoff I, Ince PG, Arzberger T, et al. Staging/typing of Lewy body related α-synuclein pathology: a study of the BrainNet Europe Consortium. *Acta Neuropathol*. 2009;117(6):635-652.
